# OCEAN—An EXCEL add-in for ^17^O correction using a novel approximation

**DOI:** 10.1016/j.mex.2023.102529

**Published:** 2023-12-20

**Authors:** Lukas Flierl, Olaf Rienitz

**Affiliations:** Physikalisch-Technische Bundesanstalt, Bundesallee 100, Braunschweig 38114, Germany

**Keywords:** ${}^{17}$O correction, Metrology, IRMS, Stable isotopes, Uncertainty calculation, Normalization, PTB ${}^{17}$O Correction Scheme

## Abstract

In this publication, an alternative mathematical approach for ${}^{17}\text{O}$ correction is presented. It is shown comprehensively how the newly developed formulas are derived and what the differences are to the established algorithm recommended by Brand et al. Additionally, two simulations are presented, which demonstrate the performance (in terms of accuracy and precision) of the approach. Part of this work is an EXCEL Add-In, which allows users to perform ${}^{17}\text{O}$ correction, normalization and uncertainty propagation on their own. This enables the users - who are limited to measure only the masses 44 to 46 - to fully control their data evaluation.

• Improved accuracy over a wider range.
• Easy use and implementation of the scheme.
• Convenient uncertainty calculation in accordance with international standards.

## Graphical abstract

**Figure:**
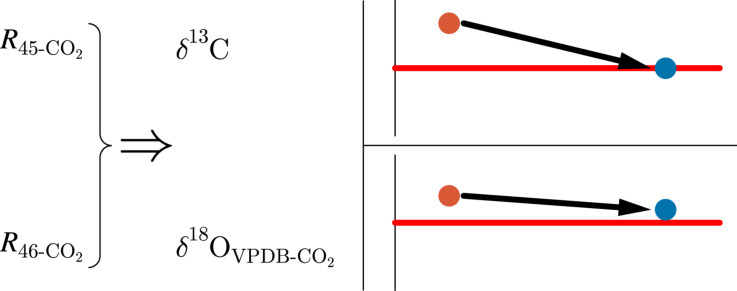

Specifications table

**Table:** 

Subject area:	Environmental Science
More specific subject area:	isotope analysis of CO${}_{2}$
Method name:	PTB ${}^{17}$O Correction Scheme
Name and reference of original method:	N. A.
Resource availability:	Microsoft EXCEL®

## Introduction

Isotope analysis of carbon dioxide (CO${}_{2}$) is an important tool in many disciplines, ranging from atmospheric sciences to paleoclimatology and several others. Usually the isotopic composition of CO${}_{2}$ is reported as a so-called δ-value on the international reference scale – the VPDB scale (VPDB = Vienna Pee Dee Belemnite). The mathematical definitions of the δ-values relevant in case of CO${}_{2}$ are:

(1) $$\delta^{13}C=\left(\frac{R_{13,\text{smp}}}{R_{13,\text{VPDB}}}-1\right)\;\;\wedge \;\delta^{18}O=\left(\frac{R_{18,\text{smp}}}{R_{18,\text{VPDB}-{\text{CO}}_{2}}}-1\right)\;$$

Where $R_{13,\text{smp}}$ and $R_{13,\text{VPDB}}$ stand for the isotope ratio ${}^{13}C$ to ${}^{12}C$ of the sample (smp) and the zero point of the scale VPDB, respectively. $R_{18,\text{VPDB}-{\text{CO}}_{2}}$ and $R_{18,\text{smp}}$ are the isotope ratios ${}^{18}$O to ${}^{16}$O of VPDB-CO${}_{2}$ and the sample, respectively. However, isotope ratios are not directly measurable using a gas mass spectrometer because of the presence of several isotopologues of the same cardinal mass (only for 44<M<49g/mol, see Table 2), therefore, the so-called ${}^{17}$O correction is necessary to derive the isotope ratios. Another reason for the ${}^{17}$O correction is that, normally only the cardinal masses 44 to 46 are recorded, the signal on mass 47 is quite low leading to insufficient accuracy and precision. Hence, only two knowns ($R_{45}$ & $R_{46}$) are available but three unknowns must be determined. This leads to an undetermined system of equations.

(2a) $$\begin{array}{c} R_{45}=R_{13}+2\cdot R_{17} \end{array}$$

(2b) $$\begin{array}{c} R_{46}=2\cdot R_{17}\cdot R_{13}+R_{17}^{2}+2\cdot R_{18} \end{array}$$

**Table 1:** Absolute isotope ratios and constants used in this publication. Uncertainties are expanded with k=2. Please note, that Brand et al. did not associate uncertainties to all of these quantities.

	value	reference
$R_{13,\mathrm{VPDB}}/$(mol mol${}^{-1}$)	0.011180(28)	[18]
$R_{17,\mathrm{VPDB}-{\text{CO}}_{2}}/$ (mol mol${}^{-1}$)	0.0003931(9)	[3]
$R_{18,\mathrm{VPDB}-{\text{CO}}_{2}}/$ (mol mol${}^{-1}$)	0.00208835	[9], [19]
λ	0.528	[20], [21], [22]
K	0.01022461	[19]

**Table 2:** All isotopologues of CO${}_{2}$ listed in increasing cardinal and molar mass.

cardinal mass	formula
44	${}^{16}$O${}^{12}$C${}^{16}$O
45	${}^{16}$O${}^{13}$C${}^{16}$O
	${}^{16}$O${}^{12}$C${}^{17}$O
46	${}^{16}$O${}^{12}$C${}^{18}$O
	${}^{16}$O${}^{13}$C${}^{17}$O
	${}^{17}$O${}^{12}$C${}^{17}$O
47	${}^{16}$O${}^{13}$C${}^{18}$O
	${}^{17}$O${}^{12}$C${}^{18}$O
	${}^{17}$O${}^{13}$C${}^{17}$O
48	${}^{18}$O${}^{12}$C${}^{18}$O
	${}^{17}$O${}^{13}$C${}^{18}$O
49	${}^{18}$O${}^{13}$C${}^{18}$O

For the sake of simplicity in the last two equations the subscripts were omitted (like smp or VPDB), but they will be reintroduced if needed. $R_{45}$ and $R_{46}$ are the molecular ratios, meaning the ratio of the isotopologues of cardinal mass 45 to mass 44, see Table 2. In order to solve these equations, it is necessary to eliminate one of the three unknowns. Craig eliminated $R_{17}$ by setting up a relation between $R_{17}$ and $R_{18}$ [1].

(3) $$R_{17}=\frac{R_{17,\mathrm{std}}}{R_{18,\mathrm{std}}^{\lambda }}\cdot R_{18}^{\lambda }=K\cdot R_{18}^{\lambda }$$

$R_{17,\mathrm{std}}$ and $R_{18,\mathrm{std}}$ are the oxygen isotope ratios of the original PDB material, to be precise the isotope ratios of CO${}_{2}$ evolved from PDB under well-defined conditions. λ is an empirical quantity describing the relation between $R_{17}$ and $R_{18}$ of materials linked by similar fractionation processes. Craig used in his work λ=0.5, which is a oversimplification of the occurring fractionation processes. At this time there was no data available that indicated the variability of λ and therefore λ=0.5 was a valid assumption. A detailed discussion about the correct choice of the value of λ is given by Brand et al. [2], this work follows their recommendations and will use the values given in Table 1. Note, Assonov and Brenninkmeijer [3] were the first to recommend λ=0.528. K is a proportionality constant which is nowadays calculated using the isotope ratios of VPDB-CO${}_{2}$ instead of CO${}_{2}$ evolved from PDB. Note, it must be mentioned that Craig did not use the symbol K in his paper. K was introduced by Santrock et al. [4]. Inserting relation (3) into Eqs. (2a) and (2b) leads to the following equations.

(4a) $$\begin{array}{c} R_{45}=R_{13}+2\cdot K\cdot R_{18}^{\lambda } \end{array}$$

(4b) $$\begin{array}{c} R_{46}=K\cdot R_{18}^{\lambda }\cdot R_{13}+{\left(K\cdot R_{18}^{\lambda }\right)}^{2}+2\cdot R_{18} \end{array}$$

Now Eq. (4a) can be solved for $R_{13}$ and inserted into Eq. (4b), and after rearranging it the following is obtained:

(5) $$0=R_{46}+3\cdot K^{2}\cdot R_{18}^{\left(2\cdot \lambda \right)}-2\cdot R_{45}\cdot K\cdot R_{18}^{\lambda }-2\cdot R_{18}$$

This equation has been formulated for the first time by Santrock et al. [4] Craig used a different mathematical approach. Using Eq. (5) and a specific set of parameters is usually referred to as the “SSH” algorithm [4]. Since Eq. (5) cannot be solved analytically, when λ≠0.5 Brand et al. [2] developed a linear approximation. The linear approximation is referred to below as “Brand et al. algorithm/scheme”. The Brand et al. algorithm bases on equations and approximations developed by Kaiser [5]. Kaiser showed that $\delta^{13}C$ can be expressed as:

(6) $$\delta^{13}C=\delta_{45}+\frac{2\cdot R_{17,\mathrm{VPDB}-{\text{CO}}_{2}}}{R_{13,\mathrm{VPDB}}}\cdot \left(\delta_{45}-\delta^{17}O\right)$$

Analogously to the isotopic δ values the molecular δ values, $\delta_{45}$ and $\delta_{46}$, are defined whereas here the difference of the ratios $R_{45}$ and $R_{46}$ of the sample to the ratios of reference VPDB-CO${}_{2}$ are meant, please see Eqs. (14) to (17) in the appendix. In the next step Kaiser approximated $\delta^{17}O$.

(7) $$\delta^{17}O\approx \lambda \cdot \delta_{46}+\Delta^{17}O$$

$\Delta^{17}O$ is the so-called oxygen isotope anomaly which is a measure for non-mass-dependent fractionation, note that there are different definitions and mathematical formulas, see Assonov et al. [6]. Brand et al. further simplified the approximation of $\delta^{17}O$, by assuming there is no non-mass-dependent fractionation (mathematically speaking; $\Delta^{17}O=0$). Hence, Brand et al. approximate $\delta^{13}C_{\mathrm{VPDB}}$ using:

(8) $$\delta^{13}C_{\mathrm{VPDB}}\approx \delta_{45}+\frac{2\cdot R_{17,\mathrm{VPDB}-{\text{CO}}_{2}}\left(\delta_{45}-\lambda \cdot \delta_{46}\right)}{R_{13,\mathrm{VPDB}}}$$

And $\delta^{18}O_{\mathrm{VPDB}-{\text{CO}}_{2}}$ is approximated using:

(9) $$\delta {}^{18}O_{\mathrm{VPDB}-{\text{CO}}_{2}}\approx \frac{\delta_{46}-0.0021\cdot \delta {}^{13}C_{\mathrm{VPDB}}}{0.99904}$$

Eq. (9) has been derived by Gonfiantini [7], whereas Brand et al. updated the numeric numbers by using the parameter set given in Table 1. Note, that there are no uncertainties associated with the numeric values (0.0021 and 0.99904) since their contribution to the uncertainty budget is according to Brand et al. negligible in daily lab routine. These approximations have two advantages over solving Eq. (5) iteratively. The first advantage is that it can be easily implemented in a spreadsheet, without any knowledge about numeric methods and secondly these analytical equations facilitate the calculations of the associated uncertainties by applying the GUM approach [8]. Moreover, Brand et al. have also presented approximations for the combined uncertainties $u_{c}$ associated with $\delta {}^{13}C$ and $\delta {}^{18}O_{\mathrm{VPDB}-{\text{CO}}_{2}}$. These are: $u_{c}\left(\delta {{}^{13}C}_{\mathrm{VPDB}}\right)\approx 1.07\cdot u_{c}\left(\delta_{45}\right)$ and $u_{c}\left(\delta {}^{18}O_{\mathrm{VPDB}-{\text{CO}}_{2}}\right)\approx u_{c}\left(\delta_{46}\right)$, but one has to consider that these simple approximations are only valid for pure CO${}_{2}$ gas samples and that they are not in accordance with the GUM. Another advantage is that the scheme proposed by Brand et al. is entirely formulated in terms of δ values, which are the quantities being used in daily lab routine and not absolute isotope ratios. Besides offering a simple linear approximation the work of Brand et al. added value by justifying a harmonized parameter set (see Table 1 in the original publication [2]), motivating to use this set and finally also offering a simple scheme for estimating uncertainties. All this enabled users to validate data received from commercial IRMS software in a convenient way. The algorithm proposed by Brand et al. is not the first which is completely formulated in δ values. Kaiser [5] also formulated an ${}^{17}$O correction algorithm completely in terms of δ values. The big advantage of Kaiser’s approach is that an oxygen isotope anomaly ($\Delta^{17}O$) can be considered, but it has to be estimated or measured before the $\delta^{13}$C and $\delta^{18}$O values can be calculated. But also considering $\Delta^{17}O=0\pm u\left(\Delta^{17}O\right)$ is possible, which is advantage over the work presented by Brand et al. and the here presented scheme. Besides the already above mentioned correction algorithms there are others like those published by Allison et al. [9], Werner at al. [10] or the one published by Miller et al. [11] This algorithm as well allows to consider ${}^{17}$O excess. Considering $\Delta^{17}O=0\pm u\left(\Delta^{17}O\right)$ is possible as well. The clumped isotope community often uses the harmonized parameter set proposed by Brand et al. and derives exact solutions using iterative methods, this is also referred as Brand 2010 algorithm even though the linear approximations are not used [12], [13], [14], [15]. This also includes contributions to uncertainty calculations [16], [17]. In this publication an alternative mathematical approach is presented with an improved performance compared to the algorithm presented by Brand et al.

## An alternative mathematical approach

In this publication an alternative mathematical approach to approximate Eq. (5) and thus an approximation for $\delta^{18}O_{\mathrm{VPDB}-{\text{CO}}_{2}}$ is presented. A detailed derivation of the following equation can be found in the supplement of this publication.

(10) $$\delta^{18}O\approx \frac{3R{{}_{17,\text{ref}}}^{2}-2\left(\delta_{45}+1\right)R_{45,\text{ref}}R_{17,\text{ref}}-2R_{18,\text{ref}}+\left(\delta_{46}+1\right)R_{46,\text{ref}}}{2\left(-3\lambda R{{}_{17,\text{ref}}}^{2}+\lambda \left(\delta_{45}+1\right)R_{45,\text{ref}}R{{}_{17,\text{ref}}}^{2}+R_{18,\text{ref}}\right)}$$

Please note, for the sake of simplicity “VPDB “ and “$\mathrm{VPDB}-{\text{CO}}_{2}$” were substituted the with “ref” being short for reference in the above equation. At this point it is crucial to point out that $R_{18,\mathrm{VPDB}-{\text{CO}}_{2}}$ and $R_{18,\mathrm{VPDB}}$ are not the same. The first refers to the isotope ratio (${}^{18}$O to ${}^{16}$O) of CO${}_{2}$ released from the virtual VPDB and the second is the ratio of the solid, virtual VPDB material. These two ratios are different due to the fractionation occurring during the acid digestion [23]. The main difference between the approximation used by Brand et al. and the newly developed approximation is that Brand et al. approximate $\delta^{17}O$, while in this work Eq. (5) is approximated. Both algorithms have in common, that a statistical distribution of all isotopes and the absence of non-mass-dependent fractionation ($\Delta^{17}O=0$, or the absence of a ${}^{17}$O excess) is assumed. In a recent publication Assonov [24] showed that by using Eq. (3), with λ=0.528, $\delta^{13}$C is only biased a little bit, in the most cases ±0.01 ‰. The bias can be neglected since it is smaller than the achievable best uncertainties for real life samples. Assonov’s study supports the underlying assumption that $\Delta^{17}$O = 0 is valid in many cases. $\delta_{45}$ and $\delta_{46}$ denote the molecular δ values relative to VPDB-CO${}_{2}$. Two big advantage of this approximation are that it can be easily implemented in a spreadsheet and it allows to consider the uncertainties of all input quantities in a uncertainty propagation. Another advantage is that, if the recommendations for the absolute isotope ratios of VPDB-CO${}_{2}$ change there will be no inconsistencies. This is not the case for the approximation presented by Brand et al.  since the occurring numeric values were calculated from the recommended isotope ratios of VPDB-CO${}_{2}$. Without any approximation $\delta {}^{13}C$ can also be expressed in terms of $\delta_{45}$ and $\delta_{46}$.

(11) $$\delta {}^{13}C=\frac{\left(\delta_{45}+1\right)R_{45,\text{ref}}-2K{\left(\left(\delta_{18}+1\right)R_{18,\text{ref}}\right)}^{\lambda }}{R_{13,\text{ref}}}-1$$

Please note two things: First, by entering the result of Eq. (10) in Eq. (11) $\delta {}^{13}C$ is approximated, but Eq. (11) in it self is exact and not an approximation. Second, note the correct usage of values in expressed in terms of ‰, for instance 50 ‰ are $50.10^{-3}$ and the latter must be used in the equations presented above. Also here we replaced “VPDB-CO${}_{2}$” with “ref” for the sake of simplicity. In order to facilitate the use of the proposed equations an EXCEL Add-in file is provided as part of the supplement accompanying this publication. The Add-in is called OCEAN, being short for **o**xygen-17 **c**orrection **e**xcel **a**dd-i**n**. This Add-in not only allows to perform the ${}^{17}$O correction also there two separate functions to calculate the uncertainties associated with $\delta {}^{13}C$ and $\delta^{18}O$, respectively. The uncertainty evaluation follows the recommendations given in [25]. Furthermore, the user has the opportunity to use custom values for the three parameters λ, $R_{18,{\mathrm{VPDB}-\text{CO}}_{2}}$ and $R_{17,{\mathrm{VPDB}-\text{CO}}_{2}}$ and their associated uncertainties. By default, the values given in Table 1 are used. Comprehensive examples are given in the supplement. At this point it is worth to mention other tools allowing ${}^{17}$O correction like a web-based application developed by Verkouteren et al. [26], a web-based application and a python package both developed by Daëron [17]. The webtool published by Verkouteren et al. (http://www.nist.gov/widps-co2) also provides the use of custom parameters and also offers the opportunity to perform a single or two-pint normalization. The same features are offered by the python package developed by Daëron. Note, our Add-in does not support clumped isotope calculations, users interested in such calculations are referred to the work of Daëron.

## Comparison with the established scheme

In this section the newly developed approximation (PTB algorithm/scheme) is compared with the established one recommended by Brand et al. as well as the numeric solution. Note, that mass spectrometer software packages (like Isodat) use a numerical method for the ${}^{17}$O correction. To assess the performance in terms of accuracy a simulation was performed and to study the influence on the uncertainties of the newly developed scheme numbers of real measurements were used.

**Fig. 1:**
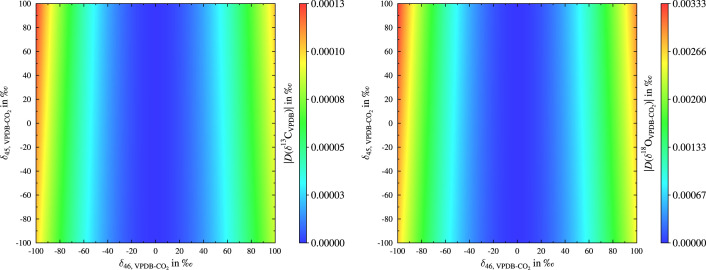
On the left, deviation of calculated $\delta^{13}C$ values from the exact solution using the newly developed scheme. On the right, deviation of calculated $\delta^{18}O$ values from the exact solution using the newly developed scheme.

**Fig. 2:**
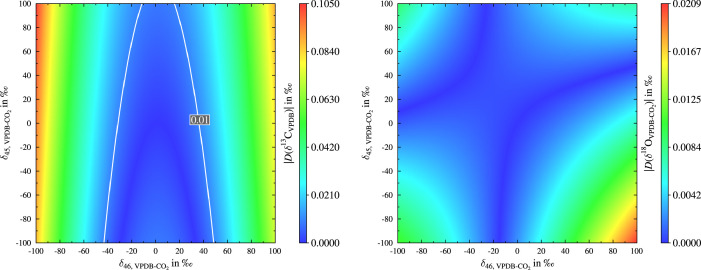
On the left, deviation of calculated $\delta^{13}C$ values using the Brand et al. scheme from the exact solution. On the right, deviation of calculated $\delta^{18}O$ from the exact solution values using Brand et al. scheme. To the left plot white contour lines were added, which facilitate to see when $|D_{\mathrm{Brand}}\left(\delta^{13}C\right)|$ is larger than 0.01 ‰.

For the simulation the molecular δ values ($\delta_{45}$ and $\delta_{46}$) were varied from -100 ‰ to 100 ‰ in 0.5 ‰ steps and for each of the 160801 tuples the $\delta^{13}C$ and $\delta^{18}O$-value were calculated using the PTB scheme, the scheme recommended by Brand et al. and the so-called Newton-Raphson method for solving Eq. (5) numerically. The values obtained by the Newton-Raphson method were considered to be the true ones. Finally, the differences between the δ values obtained from the PTB algorithm and Brand et al. scheme as well as the true (or exact) values were calculated, e.g., see Eq. (12). The results of the simulation are presented in Figs. 1 and 2. The comparison of the four plots shows, that both algorithms perform well over quite a large range, but for the algorithm presented by Brand et al. in some extreme cases the deviation from the exact values are in the same order of magnitude like the maximum achievable precision (≈0.01 ‰ [10], [27] in case of $\delta {}^{13}C$) or higher and hence the algorithm presented by Brand et al. could lead in some rare cases to results differing significantly from the true results. Please note the contour lines, which have been added to the left plot in Fig. 2. Values within the white lines are lower than 0.01 ‰. The maximum value of $D_{\mathrm{PTB}}\left(\delta^{13}C\right)$ is only $1.3.10^{-4}$ ‰ and hence insignificant. In the case of both δ values, using the PTB algorithm leads to much lower deviations from the exact results over the whole range. Please note the different colour bars. Admittedly, the extreme cases are probably far off from the usually measured samples, but this simulation should just demonstrate the high performance of the PTB algorithm.

**Fig. 3:**
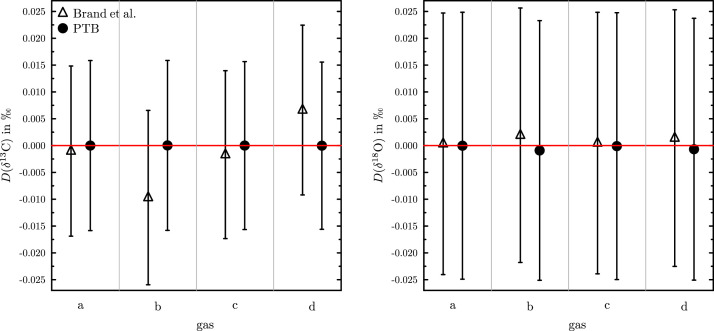
Comparison of the deviation D from the exact value. Left side $\delta {}^{13}C$ and right side $\delta^{18}O$, in both cases the PTB algorithm and the algorithm presented by Brand et al. were used. The horizontal red line represents the ideal case with D=0. The error bars represent the expanded uncertainties with k=2. (For interpretation of the references to colour in this figure legend, the reader is referred to the web version of this article.)

In a second test also the achievable uncertainties were assessed. Real life data from four different gases (gas a to d) were used. These samples were distributed during the joint CCQM (Consultative Committee for Amount of Substance: Metrology in Chemistry and Biology) pilot study P204 [28] of the IRWG and GAWG organized by the BIPM (International Bureau of Weights and Measures) and the IAEA (International Atomic Energy Agency). For the assessment the $\delta_{45}$ and $\delta_{46}$ (both relative to VPDB-CO${}_{2}$) were used. The numbers can be found in the supplement. The $\delta {}^{13}C$ and $\delta^{18}O$ values for the gases were calculated using the IUPAC recommendation, the newly developed approximation and the numeric solution (again using the Newton–Raphson method). Additionally, the uncertainties associated with the δ-values obtained by the algorithm presented by Brand et al. and PTB algorithm were calculated. The results for all four gases are summarized in Fig. 3 and in a table in the supplement of this paper. The red line in the two graphs depicts the exact solution. It can be easily seen that using PTB’s approach leads to values closer to the exact solution. This is especially the case for the $\delta {}^{13}C$ values: The more negative they are the more the values obtained using the approximation presented by Brand et al. deviate from the exact solution. Please note, that the uncertainties associated with the exact solution were not calculated, since the agreement was obvious. Though the difference between the values obtained using the established and the PTB algorithm is not significant, but it may be important when results from different labs are compared. Figure 3 shows that the uncertainties are about the same, meaning the PTB algorithm does not artificially alter the associated uncertainties. In conclusion it can be summarized that in terms of accuracy the PTB algorithm performs better and in terms of precision as well as the algorithm presented by Brand et al.

(12) $$D_{x}\left(\delta^{13}C\right)=\delta^{13}C_{x}-\delta^{13}C_{\mathrm{num}}\;,\text{with}\;x\in \left\{\mathrm{PTB},\mathrm{Brand}\right\}$$

## Scale calibration/normalization

${}^{17}$O correction is not the only step in data evaluation, since measurements directly against the virtual material VPDB is not possible and therefore the sample is usually measured against a working standard (ws). The values $\delta_{45\mathrm{smp},\text{ws}}$ and $\delta_{46\mathrm{smp},\text{ws}}$ must be converted relative to VPDB-CO${}_{2}$ ($\delta_{45\mathrm{smp},\text{VPDB}-{\text{CO}}_{2}}$ and $\delta_{46\mathrm{smp},\text{VPDB}-{\text{CO}}_{2}}$) and in order to correct also for scale contracting effects usually one or more reference materials (which cover a broad δ-value range) are measured against the ws in the same campaign. After this step, which is often called normalization or calibration, the ${}^{17}$O correction can follow. If only one reference material (rm) is used $\delta_{x\mathrm{smp},\text{VPDB}-{\text{CO}}_{2}}$ (x∈{45,46}) can be calculated using the following equation [26]. Note, single-point does not correct for scale contracting effects like cross contamination [29] and it is only mentioned here and part of the EXCEL Add-in for the sake of completeness and enabling users also to reevaluate older data sets.

(13) $$\delta_{x\mathrm{smp},\text{VPDB}-{\text{CO}}_{2}}=\frac{\left(\delta_{x\mathrm{smp},\text{ws}}+1\right)\left(\delta_{x\mathrm{rm},\text{VPDB}-{\text{CO}}_{2}}+1\right)}{\delta_{x\mathrm{rm},\text{VPDB}-{\text{CO}}_{2}}+1}-1$$

If two or more reference materials are used, a linear regression ($y=a_{1}\cdot x+a_{0}$) is fitted. The values on the x-axis are the δ-values relative to the working standard and the values on the y-axis are the certified values relative to VPDB-CO${}_{2}$. The fit parameters allow to perform the normalization. Paul et al. [30] gave a comprehensive overview of the above mentioned procedures and a modified version of the single-point normalization. Since normalization is part of the daily lab routine, we added EXCEL functions for single and multi-point normalization to our EXCEL Add-in. The function for the single-point normalization uses Eq. (13). For the multi-point normalization a linear regression is performed using the so-called total least squares method [31]. The method considers the uncertainties in both directions (y and x) and is therefore more suitable for the normalization. Furthermore, a function was implemented to calculate the uncertainty associated with the normalized value. Comprehensive examples are given in the supplement.

## Conclusion

An alternative mathematical approach for ${}^{17}$O correction was presented. This algorithm combines the advantages of an exact solution and the approximation by Brand et al. Specifically, it offers high precision and an explicit solution, which simplifies uncertainty propagation. However, the mathematics of the approximation used are slightly more complex, but still manageable (for example, in a spreadsheet) and users are rewarded with a higher precision, even in extreme cases. To facilitate the use of the new approach, an EXCEL Add-In file is provided as a supplement to this publication for the convenience of potential users. The presented EXCEL Add-In allows to also correctly consider the uncertainties associated with $R_{13,\mathrm{VPDB}}$, $R_{17,\mathrm{VPDB}-{\text{CO}}_{2}}$ and $R_{18,\mathrm{VPDB}-{\text{CO}}_{2}}$. In a simulation, it was shown that the PTB algorithm calculates values that are closer to numerical solutions than the algorithm presented by Brand et al. does. In a second example, the impact when real data is used was shown. Especially, when the sample is depleted in ${}^{13}$C, the PTB algorithm performs better especially over a larger delta range. Therefore, it can be concluded that the newly developed approximation is a competitive alternative. Additionally to the ${}^{17}$O correction, the presented EXCEL Add-in contains also functions for single or multi-point normalization and functions for uncertainty propagation for these two calibration strategies.

## CRediT authorship contribution statement

**Lukas Flierl:** Writing – original draft, Conceptualization, Visualization, Software. **Olaf Rienitz:** Writing – review & editing, Visualization.

## Declaration of Competing Interest

The authors declare that they have no known competing financial interests or personal relationships that could have appeared to influence the work reported in this paper.

## Data Availability

Data will be made available on request.

## Appendix

Mathematical definitions of $R_{45}$, $R_{46}$, $\delta_{45}$ and $\delta_{46}$:

(14) $$R_{45}=\frac{I\left({}^{16}O^{13}C^{16}O\right)+I\left({}^{16}O^{12}C^{17}O\right)}{I({}^{16}O^{12}C^{16}O)}$$

(15) $$R_{46}=\frac{I\left({}^{16}O^{12}C^{18}O\right)+I\left({}^{16}O^{13}C^{17}O\right)+I\left({}^{17}O^{12}C^{17}O\right)}{I({}^{16}O^{12}C^{16}O)}$$

(16) $$\delta_{45}=\frac{R_{45,\mathrm{smp}}}{R_{45,\mathrm{VPDB}-{\text{CO}}_{2}}}-1$$

(17) $$\delta_{46}=\frac{R_{46,\mathrm{smp}}}{R_{46,\mathrm{VPDB}-{\text{CO}}_{2}}}-1$$

Please note, I(y) denotes the ion intensity resulting from the corresponding isotopologue y.

## Appendix B Supplementary materials

Supplementary Data S1 Supplementary Raw Research Data. This is open data under the CC BY license http://creativecommons.org/licenses/by/4.0/ Supplementary Data S1
